# Anti-inflammatory Trained Immunity Mediated by Helminth Products Attenuates the Induction of T Cell-Mediated Autoimmune Disease

**DOI:** 10.3389/fimmu.2019.01109

**Published:** 2019-05-21

**Authors:** Shauna M. Quinn, Kyle Cunningham, Mathilde Raverdeau, Robert J. Walsh, Lucy Curham, Anna Malara, Kingston H. G. Mills

**Affiliations:** School of Biochemistry and Immunology, Trinity Biomedical Sciences Institute, Trinity College Dublin, Dublin, Ireland

**Keywords:** trained immunity, innate immune memory, macrophages, autoimmune disease, EAE, Th17 cells, helminth parasite

## Abstract

Recent studies have suggested that the innate immune system can display characteristics of immunological memory and this has been called “innate immune memory” or “trained immunity.” Certain fungal products have been shown to induce epigenetic imprinting on monocytes/macrophages that results in heightened inflammatory responses to subsequent stimuli. Here we report that innate immune cells can be trained to be more anti-inflammatory following exposure to products of a helminth pathogen. Macrophages trained *in vitro* with *Fasciola hepatica* total extract (FHTE) had enhanced IL-10 and IL-1RA, but reduced TNF production upon re-stimulation with FHTE or TLR ligands and this was reversed by inhibitors of DNA methylation. In contrast, macrophages trained with β-glucan or Bacillus Calmette–Guérin had enhanced TNF production upon re-stimulation with Pam3cys or LPS. Furthermore, FHTE-trained macrophages had enhanced expression of markers of alternative activated macrophages (AAM). Macrophages from mice treated with FHTE expressed markers of AAM and had heightened IL-10 and IL-1RA production in response to FHTE or TLR ligands and had suppressed TNF and IL-12p40 production. Macrophages from mice treated with FHTE had reduced APC function and inhibited IL-17 production and the encephalitogenic activity of T cells in the experimental autoimmune encephalomyelitis (EAE) model. In addition, mice pre-treated with FHTE were resistant to induction of EAE and this was associated with a significant reduction in IL-17-producing γδ and CD4 T cells infiltrating the CNS. Our findings reveal that cells of the innate immune system can be trained *in vitro* or *in vivo* to be more anti-inflammatory by exposure to helminth products and this protects mice against the induction of a T cell-mediated autoimmune disease.

## Introduction

Until recently, immunological memory was thought to be confined to antigen-specific T and B cells. However, this has recently been challenged by studies showing that innate immune responses in plants and invertebrates, which lack an adaptive immune system, can mount resistance to reinfection (1–3). In plants, epigenetic changes can lead to the priming of genes encoding host defense molecules; innate immune cells respond more rapidly and robustly following re-exposure, a process known as systemic acquired resistance (4). More recently, training of the innate immune system has also been described in vertebrates. Netea and colleagues have demonstrated that human monocytes exposed to β-glucan or the bacille Calmette-Guerin (BCG) vaccine have enhanced capacity to respond and produce pro-inflammatory cytokines upon re-stimulation with inflammatory agents or pathogens (5, 6). The enhanced responsiveness of the innate immune cells following training is mediated by epigenetic changes in macrophages, monocytes, and natural killer (NK) cells and is shorter lived and less specific than classical immunological memory of the adaptive immune system (7, 8). This concept, known as “innate immune memory” or “trained immunity,” although controversial and against the original dogma that only the adaptive immune system has memory, is gaining acceptance in the immunology community (7, 8).

Most of the studies on trained immunity to date have focused on pro-inflammatory or heightened effects of training the innate immune system (5, 6). However, the innate immune system can also mediate anti-inflammatory responses, therefore it is possible that following exposure to appropriate cues the innate immune system can be trained to be anti-inflammatory and less responsive to inflammatory stimuli. Because of their known ability to suppress immune responses helminth parasites are likely to be a useful source of products that drive anti-inflammatory trained immunity. Indeed, it is well established that helminth-induced immunosuppression is mediated in part by modulation of innate inflammatory immune responses (9, 10), as well as the generation of regulatory immune responses, especially the induction of regulatory T (Treg) cells (11, 12). In addition to subverting host protective immunity against the parasite, modulation of innate and adaptive immunity during helminth infection may also be beneficial to the host through suppression of immune responses that are pathogenic in certain immune-mediated diseases, including allergy, and autoimmunity (13). Several studies have reported that treatment of mice with helminth-derived products can modulate innate and adaptive immune responses that mediate allergy and autoimmune diseases in animal models (14–18). However, live helminth therapy has shown variable effects in the treatment of autoimmune diseases in human clinical trials (19–22). Nevertheless, epidemiological studies have revealed a significantly lower incidence of allergy and autoimmune diseases in rural areas of developing countries with a high prevalence of parasite infections (23, 24). Therefore, it is possible that helminth infections, while not necessarily effective at treating ongoing immune-mediated diseases, may be an important environmental influence in preventing the development of allergy or autoimmunity.

This study was designed to examine the hypothesis that the innate immune system can be trained to be less responsive to inflammatory stimuli following training with appropriate anti-inflammatory molecules, such as helminth products. A secondary objective was to establish if anti-inflammatory trained immunity induced by helminth products might indirectly explain the reduced susceptibility to developing autoimmune diseases in humans infected with helminth parasites. As we and others have reported that products of the helminth parasite *Fasciola hepatica* provoke anti-inflammatory immune response (9, 10, 25), we reasoned that *F. hepatica* may be a useful source of products for inducing anti-inflammatory trained immunity. Our findings demonstrate that *F. hepatica* total extract (FHTE) can train macrophages *in vitro* and *in vivo* to be more anti-inflammatory, suppressing effector Th1 and Th17 responses. Furthermore, mice pre-treated with two single injections of FHTE were resistant to the development of experimental autoimmune encephalomyelitis (EAE) and this was mediated by suppression of pathogenic T cell responses in the periphery and reduced infiltration of encephalitogenic T cells into the CNS.

## Materials and Methods

### Mice

C57BL/6 mice were bred in house from established colonies. All mice were maintained according to European Union regulations, and experiments were performed under license (AE19136/P042) from the Irish Health Products Regulation Authority with approval from the Trinity College Dublin BioResources Ethics Committee. All mice were housed under specific pathogen-free conditions. All mice within experiments were age and sex matched.

### Preparation of FHTE

Adult flukes were collected from infected bovine livers at a local abattoir (Kildare Chilling Ltd). Freshly isolated flukes were washed several times in PBS containing 100 μg/ml Penicillin-Streptomycin (PS, Sigma) to remove contaminants and cellular debris and transported to the lab. Live flukes were incubated at 5–6 worms per 3 ml in PBS/PS overnight in a cell culture incubator at 37°C and 5% CO_2._ Supernatants were removed, and the flukes were washed three times in PBS/PS before being washed twice with PBS. Supernatants were decanted after the last wash and flukes were mechanically homogenized for 5 min. The homogenate was centrifuged for 5 min at 2,000 g to remove large debris followed by centrifugation for 30 min at 15,000 g. The total soluble fraction (FHTE) was filtered through a 5 mm filter and then a 0.2 μm filter. The sterile homogenate was harvested, aliquoted and stored at −80°C. The concentration of FHTE used was based on protein content determined by the bicinchoninic acid assay. For *in vitro* studies, FHTE was used at a concentration of either 1.25% v/v (130 μg /ml) or 2.5% v/v (260 μg /ml). For *in vivo* studies, each mouse was injected with 50 μg of FHTE in 200 μl (250 μg/ml) of PBS.

### Generation of Bone Marrow-Derived Macrophages (BMDMs)

BMDMs were generated from C57BL/6 mice. Bone marrow was flushed from the bones using a 25G needle attached to a 20 ml syringe containing RPMI medium and cell clusters were disrupted by aspirating the cell suspension through a 19G needle. The single cell suspension was centrifuged at 300 g for 5 min before being resuspended in 2 ml of ammonium chloride lysis solution for 2 min in order to lyse the red blood cells. Cells were washed in RPMI and centrifuged at 300 g for 5 min and cultured in petri dishes at 1 × 10^6^ cells/ml in RPMI supplemented with 20% v/v of macrophage-colony stimulating factor (M-CSF) in the form of supernatants obtained from culture of the L929 cell line. M-CSF-producing L929 cells were obtained by transfection with a plasmid encoding murine M-CSF. On day 3, additional 2 ml of L929 medium was added to each petri dish. On day 6, loosely adherent wells were removed by washing with PBS and adherent macrophages were gently scraped in RPMI, counted and seeded at the required concentration in tissue culture plates. BMDMs were allowed to adhere and rest for at least 3 h at 37°C before use. Purify of macrophages was determined to be ~95% (CD11b^+^, F480^+^, GR1^−^).

### Arginase Activity Assay

Cells were incubated in 80 μl of passive lysis buffer (Promega) containing protease inhibitors. Arginase activity of cell lysate was tested using the Quantichrom Arginase Assay Kit (Bioassay Systems). A urea working standard (1 mM) was made and 25 μl of the urea working standard solution was then added to a well. Next, 20 μl of each sample was added to each appropriate well, with one well containing H_2_O and another as a blank well to be left substrate free. Five microliter of arginase substrate buffer was added to each sample, bar the blank well. Arginase activity was determined by the quantity of urea produced in a 2 h period. Eighty microliter of urea reagent containing 1:1 of reagent A and reagent B was added to all wells. Finally, the substrate buffer was added to the blank well. This method utilizes a chromogen that forms a colored complex specifically with urea produced in the arginase reaction. The intensity of the color is directly proportional to the arginase activity in the sample. The OD values were determined by measuring the absorbance at 430 nm using a microtiter plate reader. Arginase activity was calculated in units/L only or LPS (100 ng/ml) for 24 h. Supernatants were collected 24 h after re-stimulation and the concentration of cytokines quantified by ELISA.

### Treatment of Mice

C57BL/6 mice were injected i.p or s.c. with PBS, FHTE (50 μg) or LPS (10 μg) on d−21 and d−7. On d 0 either peritoneal exudate cells (PEC) were isolated from the peritoneal cavity as described below or EAE was induced in mice.

### Isolation of PEC by Peritoneal Lavage

Mice were sacrificed by asphyxiation with CO_2_ and the peritoneum was exposed by removing the skin in the abdomen. Cold PBS (6 ml) was injected into the peritoneal cavity using a 27G needle. The mouse was gently shaken to detach any adherent peritoneal cells, and 5 ml were removed with a 19G needle. PEC were placed on ice in a 15 ml Falcon tube to prevent adherence of macrophages to the plastic. Cells were counted and cultured at the required concentration in tissue culture plates.

### Purification of F4/80^+^ CD11b^+^ or Small Peritoneal Macrophages by FACS

Freshly isolated PEC were washed and resuspended in a volume of 1 ml. The cells were incubated with Fc block (BD Biosciences; 5 μl/1 ml) for 10 min to prevent non-specific binding. For F4/80^+^ CD11b^+^ macrophages, PEC were surface stained with 1 μl per 5 × 10^6^ cells of fluorescent antibodies directed against CD11b-APC-eFluor780 and F4/80-PECy5 (eBioscience, UK) for 15 min at room temperature (RT) in the dark. For small peritoneal macrophages (SPM), PEC were surface stained with 1 μl per 5 × 10^6^ cells of fluorescent antibodies directed against CD11b, F4/80 and MHC II-FITC (eBioscience, UK) for 15 min at RT in the dark. Cells were washed and resuspended in 1 ml of FACS buffer and sorted by flow cytometry. SPM were identified as CD11b^+^ F4/80^low^ MHCII^high^ cells.

### MOG-Specific T Cell Responses

MOG-specific T cells were generated in mice by s.c immunization with MOG_35−55_ peptide (100 μg; Genscript) and CFA (Chondrex). After 10 d, spleen and LN cells (70% spleen and 30% LN) were cultured with MOG for 72 h. Supernatants were removed and the concentrations of IL-17A, GM-CSF, and IFN-γ determined by ELISA (R&D Systems and BD Biosciences).

### APC Function of Macrophages

Macrophages were trained with FHTE *in vivo* before being co-cultured at different ratios with spleen and LN cells (70% spleen and 30% LN) in the presence of the autoantigen myelin oligodendrocyte glycoprotein (MOG; 100 μg/ml), from mice injected with MOG (100 μg) emulsified in CFA 10 d earlier. After 72 h, supernatants were collected and the concentration of IL-17A alone or IL-17A and IFN-γ determined by ELISA. To determine the APC activity of FHTE-trained macrophages, purified CD4 T cells from mice immunized with MOG (100 μg) emulsified in CFA 10 d earlier were stimulated with MOG (100 μg/ml) in the presence of irradiated macrophages (ratio of 5 CD4 T cells to 1 macrophage) acting as APCs from FHTE-treated or PBS-injected mice. After 72 h, supernatants were collected and the concentration of IL-17A and IFN-γ determined by ELISA.

### Induction and Assessment of EAE

Active EAE was induced by s.c immunization with MOG_35−55_ peptide (100 μg) and CFA and i.p. injection with pertussis toxin (PT) (100 ng; Kaketsuken) as previously described (26). PT is required for active induction of EAE as it enhances migration of T cells across the blood-brain-barrier. Disease severity was assessed according to percentage weight change and typical clinical scores as previously described (26). Spleen and LN cells were isolated on d 3 or d 7 of EAE and examined by FACS analysis or re-stimulated *ex vivo* on d 7 with medium or MOG_35−55_ (12.5–50 μg/ml) for 72 h. Supernatants were removed and the concentrations of IL-17A, GM-CSF and IFN-γ determined by ELISA (R&D Systems and BD Biosciences).

### Treatment of Mice With Trained Macrophages

C57BL/6 mice were injected i.p. with PBS or FHTE (50 μg) on d−21 and d−7. On d 0 cells were isolated from the peritoneal cavity. F4/80^+^CD11b^+^ macrophages were purified by FACS as described above and 2 × 10^6^ cells were injected i.v. into mice on d 8 and d 15 of EAE.

### Isolation of Mononuclear Cells From the CNS

Mice were sacrificed and perfused with 20 ml of PBS before removal of the brain. Organs were lysed in 1 ml RPMI using a tissue lyser (Qiagen). Tissue homogenates were resuspended in 5 ml 40% isotonic Percoll and layered over 5 ml 70% isotonic Percoll before centrifugation at 1,300 rpm for 20 min. Mononuclear cells were removed from the interface of the Percoll gradients, passed through a 70 μm filter and washed in RPMI. Cells were stimulated for cytokine detection by flow cytometry.

### Reverse Transcription-qPCR

RNA was extracted from BMDMs or SPM using the chloroform/isopropanol method and was reverse transcribed into cDNA using a High Capacity cDNA Reverse Transcription Kit (Applied Biosystems). RT-qPCR was performed using commercially available *arg1* (Mm00475991_m1), *mrc1* (313688), *chi3l3* (312249) and *retnla* (Mm00445109_m1) primers (Roche or ABI). RT-qPCR was performed on a PRISM7500 Sequence Detection System (ABI). The amount of each gene was determined by normalization to 18S rRNA (Mm04277571, ABI) internal control.

### Flow Cytometry

PEC or mononuclear cells isolated from the spleen, LNs (inguinal, brachial, and axillary) and CNS were incubated with live/dead stain (Invitrogen, Ireland; 1 in 500 dilution) and Fc block (BD Biosciences; 5 μl/1 ml), followed by surface staining with fluorochrome-conjugated anti-mouse antibodies for various markers. To detect cytokines, cells were stimulated with PMA (Sigma-Aldrich; 50 ng/mL) and ionomycin (Sigma-Aldrich; 500 ng/mL) in the presence of BrefeldinA (Sigma-Aldrich; 5 μg/mL) for 4 h at 37°C. The following surface antibodies were used at a 1 in 200 dilution: CD11b-APC-eFluor780, MHCII-FITC, F4/80-PECy5, Vγ4-PECy7, CD3-APC (eBioscience, UK), CD206-BV605, PD-L2-PE, CD4-BV785, CD45-BV785 (Biolegend, UK), Ly6G-BV605, TCR-δ-FITC, Ly6C-FITC (BD Biosciences, USA). For intracellular cytokines detection, cells were fixed in 2% PFA and permeabilized with 0.5% saponin (Sigma-Aldrich, Ireland) followed by a 1 in 200 dilution staining with IL-17A-BV650 (Biolegend, UK) and IFN-γ-PE-CF594 (BD Biosciences, USA). The Foxp3/Transcription Factor Staining Buffer Set was used according to the manufacturer's protocol to detect expression of the proliferation marker Ki67-PerCP-eFluor710 (eBioscience, UK; 1 in 200 dilution) by CD4 T cells in the brain. Fluorescence minus one (FMO) was used as controls. Flow cytometric analysis was performed on an LSR Fortessa and data acquired using Diva software (BD Biosciences, USA). The results were analyzed using FlowJo software (Tree Star, USA).

### Statistical Analysis

Statistical analysis was performed using GraphPad Prism. Data were analyzed using 1- or 2-way ANOVA followed by the multiple comparison tests, or unpaired *t*-test as appropriate. Repeated measures ANOVA followed by Sidak post-test was used to determine the statistical differences between groups in a time course. Error bars indicate the mean ± standard deviation (SD) or mean ± standard error of the mean (SEM) as indicated in the individual figure legends. Statistical significance was considered for *p-*values less than 0.05.

## Results

### FHTE Induces Anti-inflammatory Responses and Suppresses LPS-Induced Pro-Inflammatory Responses in BMDMs

**Figure 1:**
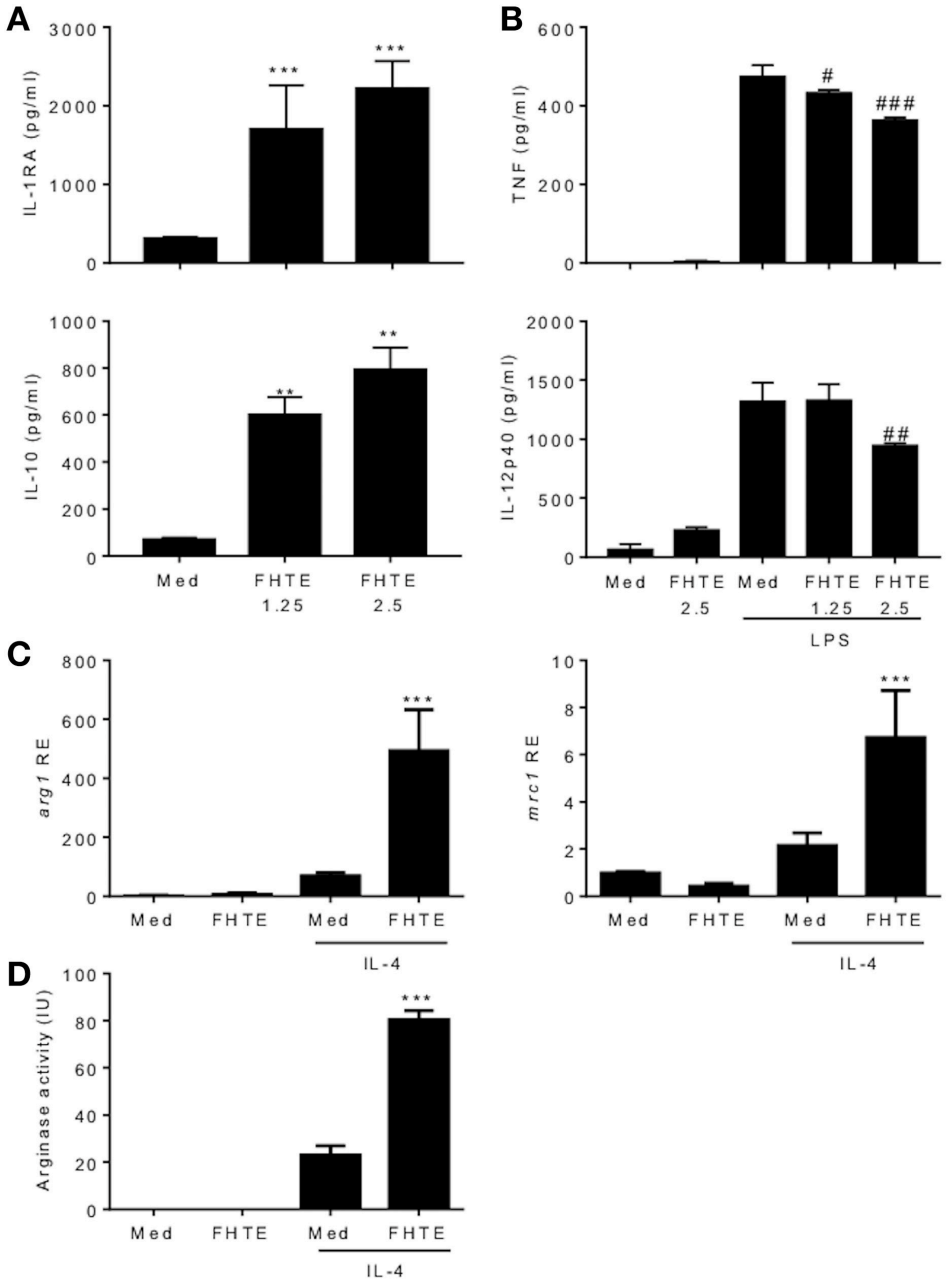
FHTE promotes IL-1RA and IL-10 and suppresses LPS-induced TNF and IL-12p40 production by macrophages. **(A)** BMDMs from C57BL/6 mice were stimulated with 1.25 or 2.5% v/v FHTE for 24 h. IL-1RA and IL-10 production was quantified in the supernatants by ELISA. **(B)** BMDMs were stimulated with FHTE (1.25 or 2.5% v/v), LPS (100 ng/ml) or a combination of both. After 24 h TNF and IL-12p40 production was measured in the supernatants. Results are mean (±SD) for triplicate culture and represent one of three independent experiments. ^**^p < 0.01, ^***^p < 0.001 vs. medium cultured cells or #*p* < 0.05, ##*p* < 0.01, ###*p* < 0.001 vs. LPS-treated cells by one-way or two-way ANOVA with Dunnett *post hoc* analysis. **(C,D)** BMDMs were cultured with 2.5% v/v FHTE, IL-4 (10 ng/ml) or a combination of both for 24 h. **(C)** Total RNA was isolated and the expression of *arg1* and *mrc1* was analyzed by RT-qPCR relative to medium cultured cells following normalization by the endogenous control 18s rRNA. **(D)** Cell lysates were tested for arginase activity. Results are mean (±SD) for triplicate culture and represent one of three independent experiments. ^***^p < 0.001 vs. IL-4-treated cells by two-way ANOVA with Dunnett *post hoc* analysis.

We have previously reported that infection of mice with *F. hepatica* resulted in recruitment and activation of regulatory DCs, macrophages and T cells (17). Here we examined the possible innate immune training effect of helminth parasites, using products of *F. hepatica*. We found that treatment of BMDMs with FHTE for 24 h induced significant IL-1RA and IL-10 production (Figure 1A), but significantly suppressed LPS-induced TNF, at both concentrations and IL-12p40 production, at the higher concentration of FHTE (Figure 1B). Macrophages are known to adopt an alternatively activated phenotype when activated in response to helminth infection through IL-10 production (27). Furthermore, IL-4-activated macrophages help to control helminth infections and induce tissue repair (28). We found that FHTE significantly enhanced mRNA expression of alternative activated macrophage (AAM) markers *arg1* and *mrc1* in IL-4-activated BMDMs (Figure 1C). In addition, treatment with 2.5% v/v FHTE, while not inducing activation of arginase alone, significantly enhanced IL-4-induced arginase activity (Figure 1D). These results demonstrate that FHTE promotes production of anti-inflammatory cytokines by macrophages and enhances IL-4-induced expression of markers of AAM.

### FHTE Trains Innate Immune Cells to be More Anti-inflammatory

**Figure 2:**
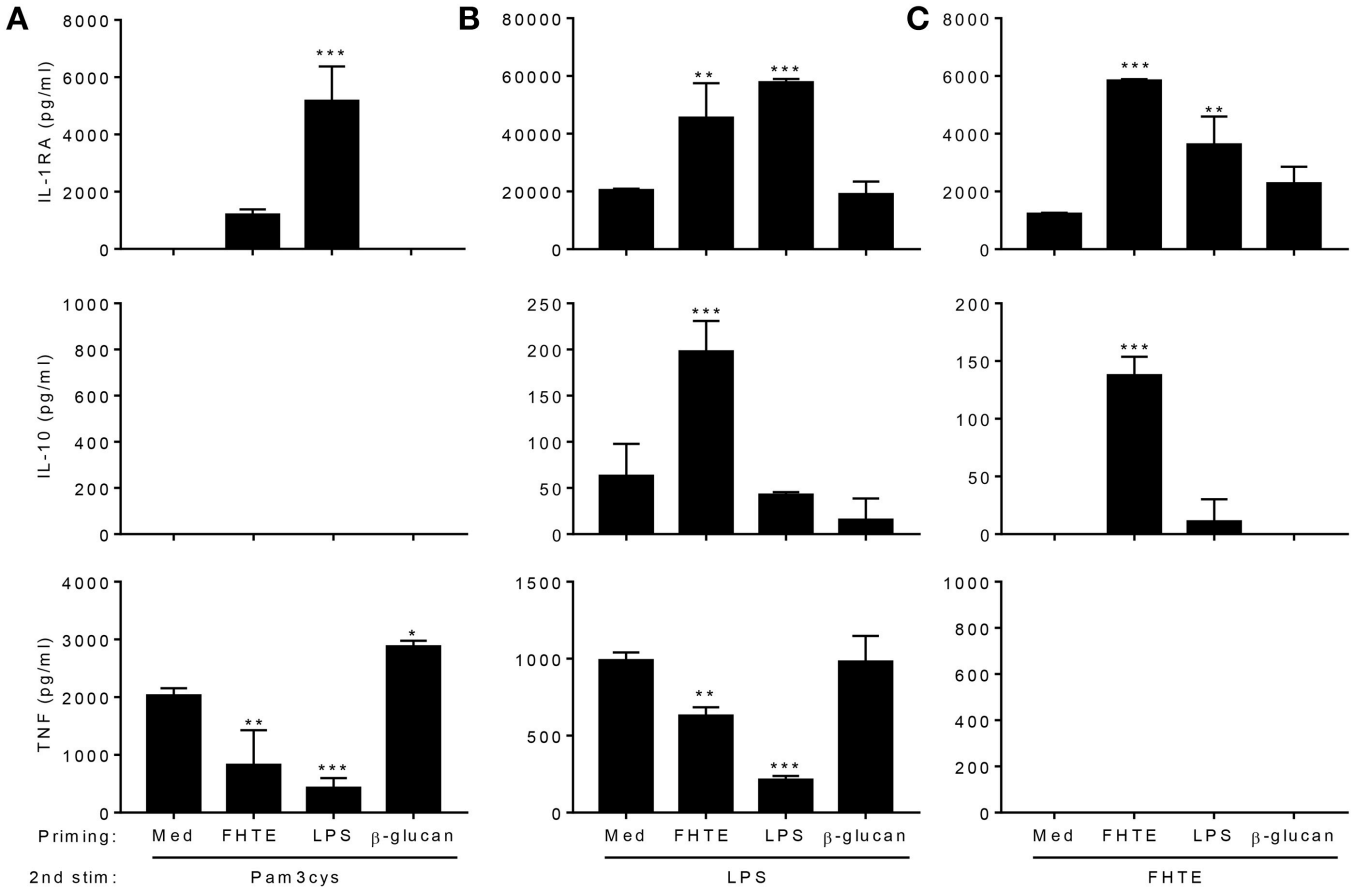
Training of macrophages with FHTE *in vitro* enhances anti- and inhibits pro-inflammatory cytokine production. **(A–C)** BMDMs were incubated with 2.5% v/v FHTE, LPS (100 ng/ml) or β-glucan (5 μg/ml). Cells were washed after 24 h and left to rest. After 3 d, cells were re-stimulated with either 2.5% v/v FHTE, LPS (100 ng/ml) or Pam3cys (10 μg/ml). Supernatants were collected 24 h after re-stimulation and the concentration of IL-1RA, IL-10 and TNF was determined by ELISA in cells re-stimulated with Pam3cys **(A)**, LPS **(B)**, and FHTE **(C)**. Results are mean ± SD and represent one of four experiments. ^*^p < 0.05, ^**^p < 0.01, ^***^p < 0.001 vs. cells cultured with medium during 1st stimulation by one-way ANOVA with Dunnett *post hoc* analysis.

We next examined the capacity of FHTE to train innate immune cells *in vitro* and compared its effect to that of LPS and β-glucan. LPS is known to induce long-term tolerance in macrophages, resulting in reduced production of pro-inflammatory mediators following re-stimulation, whereas training with β-glucan can enhance TNF and IL-6 production following re-stimulation of monocytes/macrophages with Pam3Cys (5). BMDMs were stimulated with either FHTE, LPS or β-glucan and after 24 h, cells were washed and rested for 3 d before re-stimulation with FHTE, LPS or Pam3cys. A rest period of 3 d was found to be optimum as cell viability reduced after 4 or more days of rest. We first assessed cytokines in supernatants 4 d after initial stimulation with FHTE and β-glucan, before re-stimulation, and found that IL-1RA, IL-10, and TNF concentrations were comparable to those for un-stimulated cells (Supplemental Figure 1). Following initial stimulation with LPS, IL-10 and TNF production on day 4 was also comparable to cells stimulated with medium only, however, IL-1RA production was higher in LPS-stimulated cells before restimulation when compared with medium control (Supplemental Figure 1). Basal concentrations of IL-1RA, IL-10, and TNF production from cells maintained in culture medium only for 5 d were found to be negligible (data not shown). FHTE-trained BMDMs produced higher concentrations of IL-1RA than untrained cells in response to Pam3Cys, LPS or FHTE (Figures 2A–C), as well as higher concentrations of IL-10 in response to LPS and FHTE (Figures 2B,C), but produced lower TNF than untrained cells following re-stimulation with Pam3Cys and LPS (Figures 2A,B). In contrast, BMDMs trained with β-glucan had enhanced TNF production upon re-stimulation with Pam3Cys (Figure 2A), but did not produce IL-1RA or IL-10 upon re-stimulation with FHTE, LPS or Pam3Cys (Figures 2A–C). Furthermore, training of BMDMs with LPS suppressed their ability to secrete TNF in response to Pam3Cys or LPS (Figures 2A,B), but did not enhance their ability to produce IL-10 upon re-stimulation with LPS or FHTE (Figures 2B,C). Training of BMDMs with LPS enhanced IL-1RA production upon re-stimulation with Pam3cys, LPS and FHTE (Figures 2A–C). Interestingly, no IL-10 production was detected upon restimulation with Pam3Cys after training with FHTE, LPS, or β-glucan (Figure 2A). Similarly, TNF was not detected following restimulation with FHTE, regardless of the initial training stimuli (Figure 2C). These findings suggest that FHTE can train innate immune cells non-specifically to be anti-inflammatory and this effect is distinct from that observed in LPS or β-glucan-trained cells.

### Training Effects of FHTE Are Mediated by Histone Methylation

**Figure 3:**
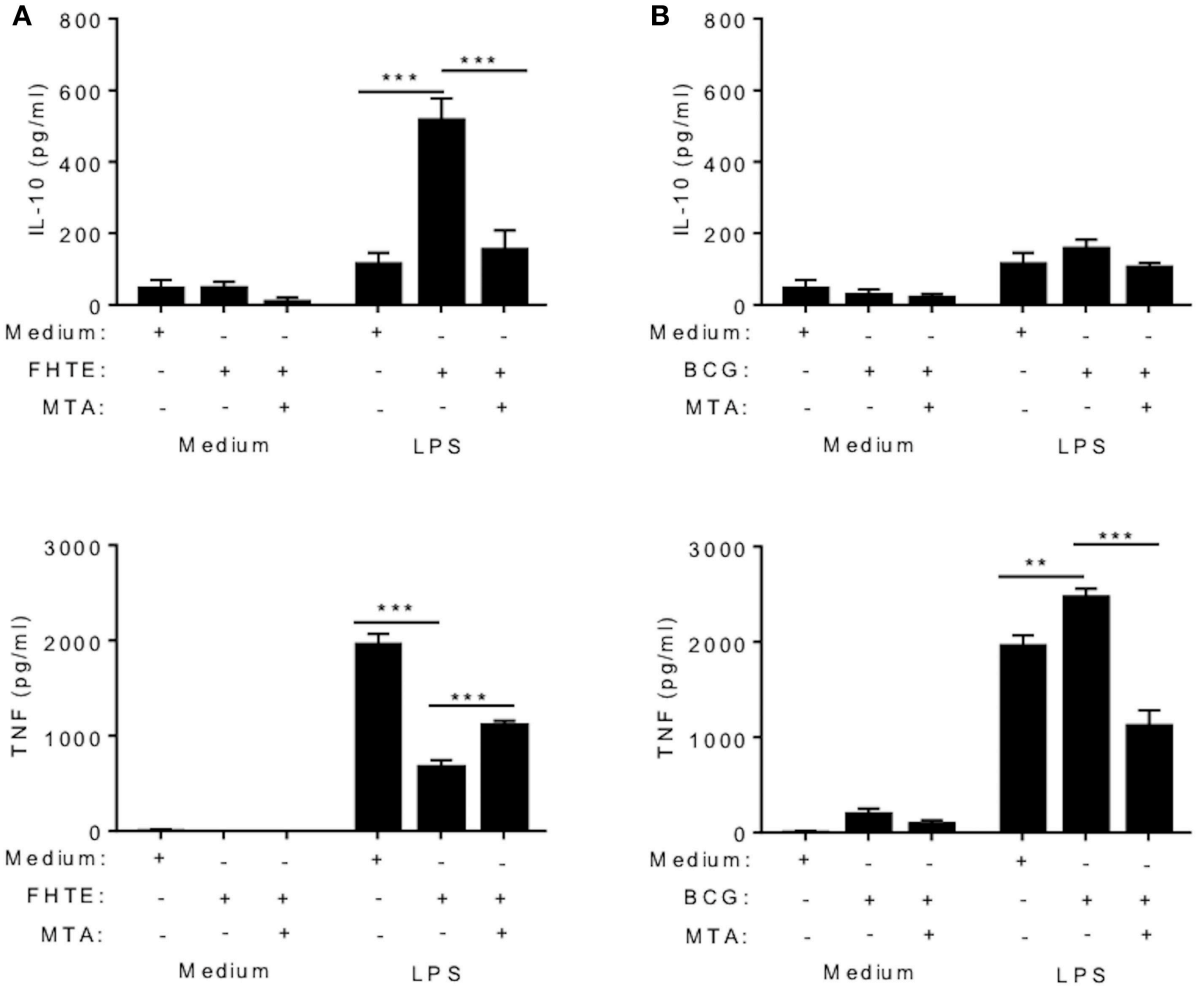
Trained immunity induced by FHTE is mediated by histone methylation. BMDMs were stimulated with methyltransferase inhibitor MTA (1 mM) for 1 h prior to treatment with FHTE (2.5% v/v) or as a control BCG (1 mM) for 24 h before being washed and rested. After 3 d, Cells were then restimulated with either medium only or LPS (100 ng/ml) for 24 h. Supernatants were collected 24 h after re-stimulation and the concentration of IL-10 and TNF was determined by ELISA in cells stimulated with FHTE **(A)** or BCG **(B)**. Results are mean ± SEM and combined from two separate experiments. ^**^p < 0.01, ^***^p < 0.001 vs. controls (BCG-trained cells that were restimulated with LPS or FHTE-trained cells that were restimulated with LPS) by one-way ANOVA with Dunnett *post hoc* analysis.

Several studies have demonstrated that trained immunity induced by β-glucan or BCG is mediated by epigenetic changes in innate immune cells resulting in heightened immune responses, characterized by enhanced proinflammatory cytokine production (5, 29, 30). Here we examined the possible role of epigenetic modifications in the anti-inflammatory training induced by FHTE. BMDMs were stimulated with methyltransferase inhibitor MTA (5′- deoxy-5′-methylthio-adenosine; 1 mM) for 1 h prior to treatment with FHTE (2.5% v/v) or as a control BCG (1 mM) for 24 h before being washed and rested for 3 d. Cells were then restimulated with either medium only or LPS (100 ng/ml) for 24 h. Consistent with the data shown in Figure 2, FHTE-trained cells produce significantly more IL-10 upon re-stimulation with LPS and this was returned to background levels by treatment with MTA (Figure 3A). Furthermore, FHTE-trained cells produce less TNF when compared with non-trained cells that were re-stimulated with LPS, and addition of MTA significantly enhanced LPS-induced TNF by FHTE-trained macrophages (Figure 3A). In contrast, BCG-trained murine macrophages produced significantly higher concentrations of TNF in response to LPS when compared with non-trained cells, and this was reduced by addition of MTA (Figure 3B), which is consistent with previous reports demonstrating a role for histone methylation in BCG induced trained immunity in human monocytes (31). These findings demonstrate that, unlike BCG which induced heighten pro-inflammatory responses following *in vitro* training, FHTE induces anti-inflammatory trained immunity and these effects appear to be mediated by histone methylation in macrophages.

### Macrophages From Mice Treated With FHTE Have an Anti-inflammatory Trained Phenotype

**Figure 4:**
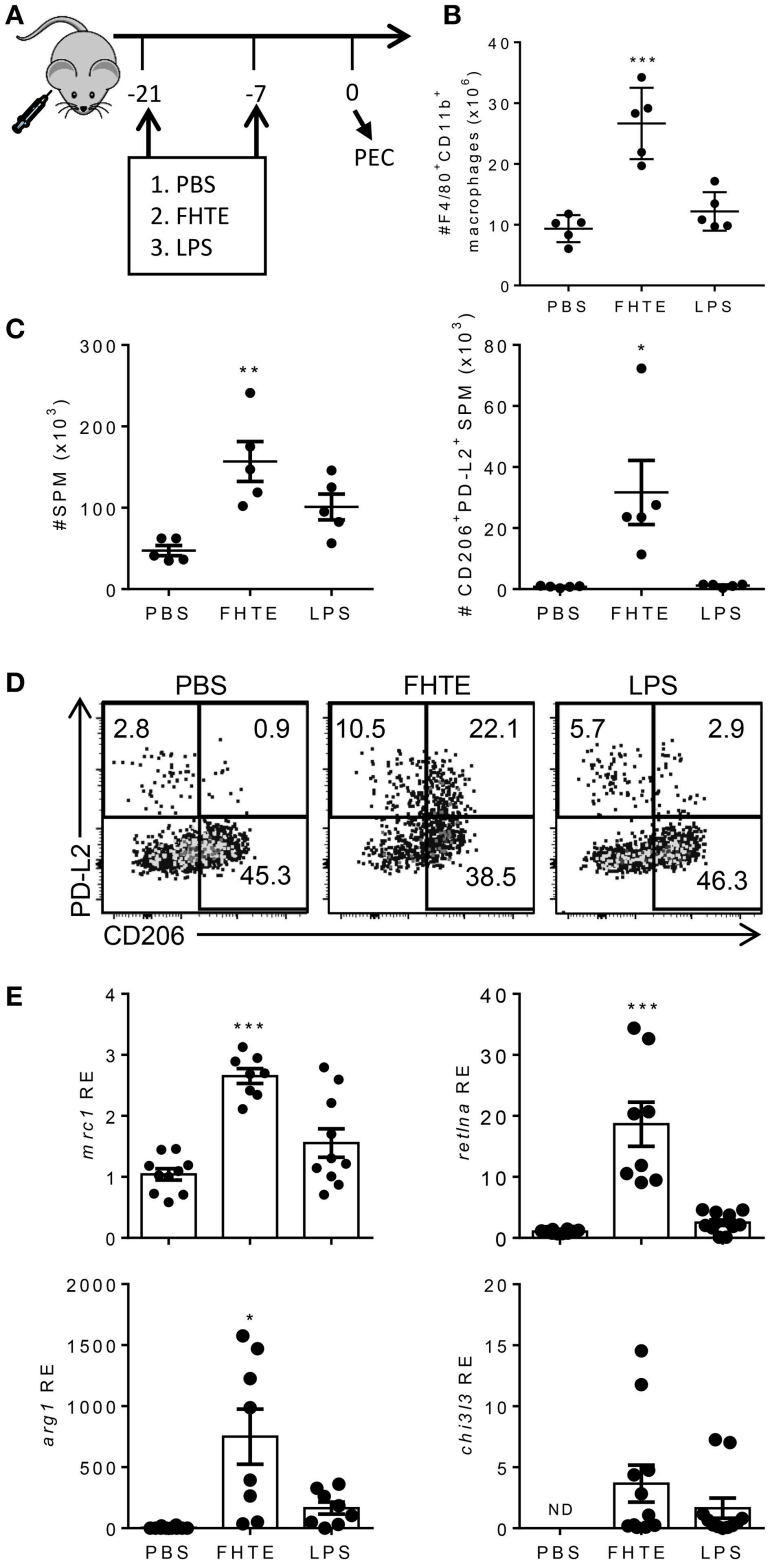
Treatment of mice with FHTE generates SPM with an AAM phenotype. C57BL/6 mice were injected i.p. with PBS, FHTE (50 μg) or LPS (10 μg) on d−21 and d−7 and on d 0 PEC cells were isolated. **(A)** Scheme of *in vivo* innate immune training. **(B)** PEC cells were stained for F4/80 and CD11b and analyzed by flow cytometry. Results are mean ±SEM (*n* = 5 or 6) and represent one of three independent experiments. Each symbol represents an individual mouse. ^***^p < 0.001 vs. PBS-injected mice by one-way ANOVA with Dunnett *post hoc* analysis. **(C,D)** PEC cells were stained for F4/80, CD11b, MHCII, CD206, and PD-L2 and analyzed by flow cytometry. Results shown are absolute number of SPM and absolute number of PD-L2^+^CD206^+^ SPM **(C)**, with representative FACS plots **(D)**. Data are mean ±SEM (*n* = 5) and represent one of two independent experiments. Each symbol represents an individual mouse. ^*^p < 0.05, ^**^p < 0.01 vs. PBS-injected mice by one-way ANOVA with Dunnett *post hoc* analysis. **(E)** SPM were FACS sorted, total RNA was extracted and the mRNA expression of *mrc1, retlna, arg1, chi3l3* was evaluated by RT-qPCR relative to PBS-injected mice following normalization by the endogenous control 18s mRNA. Results are mean ±SEM (*n* = 5–10) combined from two independent experiments. Each symbol represents an individual mouse. ^*^p < 0.05, ^***^p < 0.001 vs. PBS-injected mice by one-way ANOVA with Dunnett post hoc analysis. *ND* = not detected.

Our data suggests that FHTE can train innate immune cells *in vitro* to be more anti-inflammatory. To test if innate immune cells can be trained *in vivo*, C57BL/6 mice were injected i.p. with PBS, FHTE (50 μg) or LPS (10 μg) at 21 and 7 d before sacrifice (Figure 4A). Mice treated with FHTE had a significant increase in the number of macrophages in the peritoneal cavity when compare with PBS or LPS-treated mice (Figure 4B), indicating possible expansion of tissue resident macrophages and/or recruitment of circulating monocytes.

The peritoneal cavity contains two distinct macrophage populations, large peritoneal macrophages (LPM) and small peritoneal macrophages (SPM). LPM are tissue resident cells that make up approximately 90% of macrophages in naïve mice and express high levels of the macrophage surface markers, CD11b and F4/80. SPM are derived from blood monocytes and enter the peritoneal cavity upon stimulation or infection. Furthermore, mature SPM express high levels of MHC II, but lower levels of CD11b and F4/80 (32). Treatment of mice with FHTE resulted in an increase in the numbers of SPMs in the peritoneal cavity (Figure 4C), suggesting recruitment of monocyte-derived macrophages in response to FHTE. Helminth infections are known to trigger highly polarized type 2 immune responses, typically associated with induction of Th2 cells and increased numbers of AAMs. These AAMs are known to be derived from either proliferation of tissue resident macrophages (LPM) or recruitment of SPM to the site of injection (27). We also found increases in the frequency and absolute number of SPM that expressed CD206 and PD-L2, markers of AMMs, in mice treated with FHTE, but not in mice treated with LPS (Figures 4C,D).

To confirm the finding that treatment of mice with FHTE polarizes AAMs, we demonstrated that mRNA expression of *mrc1, retlna* and *arg1* was significantly increased in SPM in mice treated with FHTE, but not in LPS-treated mice (Figure 4E). There was also a modest increase in *chi3l3* expression when compared with PBS-injected mice. These findings demonstrate that macrophages from mice treated with FHTE are trained to have an AAM phenotype.

### Macrophages From Mice Treated With FHTE Are Anti-inflammatory, Have Reduced Capacity to act as APCs and Suppress MOG-Specific T Cell Responses That Mediate EAE

**Figure 5:**
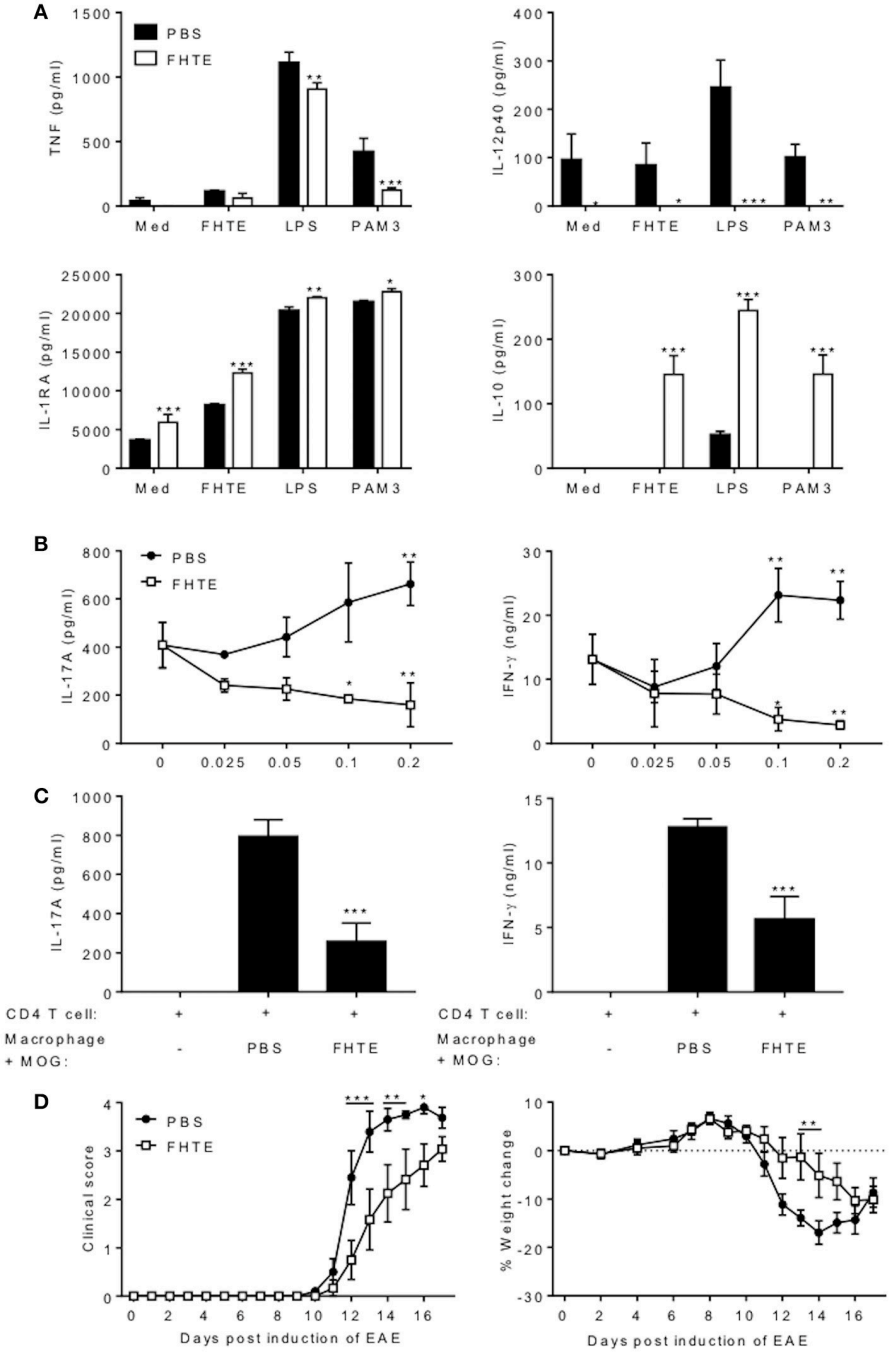
Macrophages from mice treated with FHTE are anti-inflammatory and suppress autoantigen-specific T cell responses. Six C57BL/6 mice were injected i.p. with PBS or FHTE (50 μg) on d−21 and−7 and PEC were isolated on d 0. **(A)** F4/80^+^CD11b^+^ cells were FACS sorted and re-stimulated with either 2.5% v/v FHTE, LPS (100 ng/ml) or PAM3 (10 μg/ml). Supernatants were collected 24 h after re-stimulation and the concentration of TNF, IL-12p40, IL-1RA, and IL-10 determined by ELISA. Results are mean (±SD) and represent one of two independent experiments. ^*^p < 0.05, ^**^p < 0.01, ^***^p < 0.001 vs. PBS-injected mice by two-way ANOVA with Sidak *post hoc* analysis. **(B)** FACS sorted F4/80^+^CD11b^+^ cells from 6 PBS-injected or FHTE-treated mice were co-cultured at different ratios with spleen and LN cells (70% spleen and 30% LN) in the presence of MOG (100 μg/ml), from mice injected with MOG (100 μg) emulsified in CFA 10 d earlier. After 72 h, supernatants were collected and the concentration of IL-17A and IFN-γ determined by ELISA. Results are mean (±SD) and represent one of two independent experiments. ^*^p < 0.05, ^**^p < 0.01 vs. control (no macrophages in culture) by two-way ANOVA with Sidak *post hoc* analysis. **(C)** Purified CD4 T cells from 3 mice immunized with MOG (100 μg) emulsified in CFA 10 d earlier were stimulated with MOG (100 μg/ml) in the presence of irradiated macrophages (at a ratio of 5 CD4 T cells to 1 macrophage) acting as APCs from FHTE-treated or PBS-injected mice. After 72 h, supernatants were collected and the concentration of IL-17A and IFN-γ determined by ELISA. Results are mean (±SD) and represent one of two independent experiments. ^***^p < 0.001 vs. macrophages from PBS-injected mice by one-way ANOVA with Dunnett *post hoc* analysis. **(D)** F4/80^+^CD11b^+^ macrophages from 8 PBS-injected or FHTE-treated mice were injected i.v. into mice 8 and 15 d after induction of EAE. Results are mean clinical score and percentage body weight loss. Data are mean ± SEM (*n* = 6). ^*^p < 0.05, ^**^p < 0.01, ^***^p < 0.001 vs. macrophages from PBS-injected mice by repeated measures ANOVA with Sidak *post hoc* analysis.

We next examined the function of trained macrophages from mice treated with FHTE. An examination of cytokine production revealed that macrophages from mice treated with FHTE had enhanced IL-1RA and IL-10 production but suppressed TNF and IL-12p40 production in response to re-stimulation with FHTE, LPS or Pam3Cys *ex vivo* (Figure 5A). These findings suggest that macrophages from mice treated with FHTE have been trained to be anti-inflammatory. Therefore, we examined their capacity to suppress pathogenic T cells that mediate autoimmune disease. MOG-specific Th1 and Th17 cells that mediate EAE were generated by immunization of mice with the autoantigen MOG and CFA. Ten days after immunization, spleen and LN cells (70% spleen and 30% LN) were recovered and cultured with difference ratios of macrophages from PBS or FHTE-injected mice, in the presence of MOG. MOG-induced IL-17A and IFN-γ production from spleen and LN cells was enhanced by the addition of macrophages from mice injected with PBS, but was significantly suppressed by the addition of macrophages from mice treated with FHTE. This effect was greatest at a ratio of 5 spleen/LN cells to 1 macrophage (Figure 5B).

We next examined the capacity of macrophages from mice treated with FHTE to act as APCs for MOG-specific T cells. IL-17 and IFN-γ production by purified CD4 T cells from mice immunized with MOG and CFA was significantly lower when co-cultured with MOG-pulsed macrophages (at a ratio of 5 CD4 T cells to 1 macrophage) from mice treated with FHTE compared with MOG-pulsed macrophages from PBS-treated mice (Figure 5C). These findings demonstrate that innate training with FHTE inhibits the APC function of macrophages, resulting in suppression of autoantigen-specific T cell responses.

Finally, we assessed the capacity of macrophages from mice treated with FHTE to modulate the course of disease in mice with ongoing EAE. Our hypothesis was that the anti-inflammatory macrophages might suppress cytokine production and migration of encephalitogenic T cells into the CNS. Since pathogenic T cells are first detectable in the CNS around 8–10 d after induction of EAE, we transferred macrophages on d 8 and 15. Treatment of mice with macrophages from mice treated with FHTE on d 8 and 15 of EAE significantly delayed the clinical course of EAE, as well as weight loss associated with disease (Figure 5D). These results demonstrate that macrophages from mice treated with FHTE have been trained to be anti-inflammatory, have reduced capacity to act as APC and can suppress MOG-specific T cell responses that mediate EAE.

### Pre-treatment of Mice With FHTE Attenuates EAE by Suppressing Pathogenic T Cell Responses

**Figure 6:**
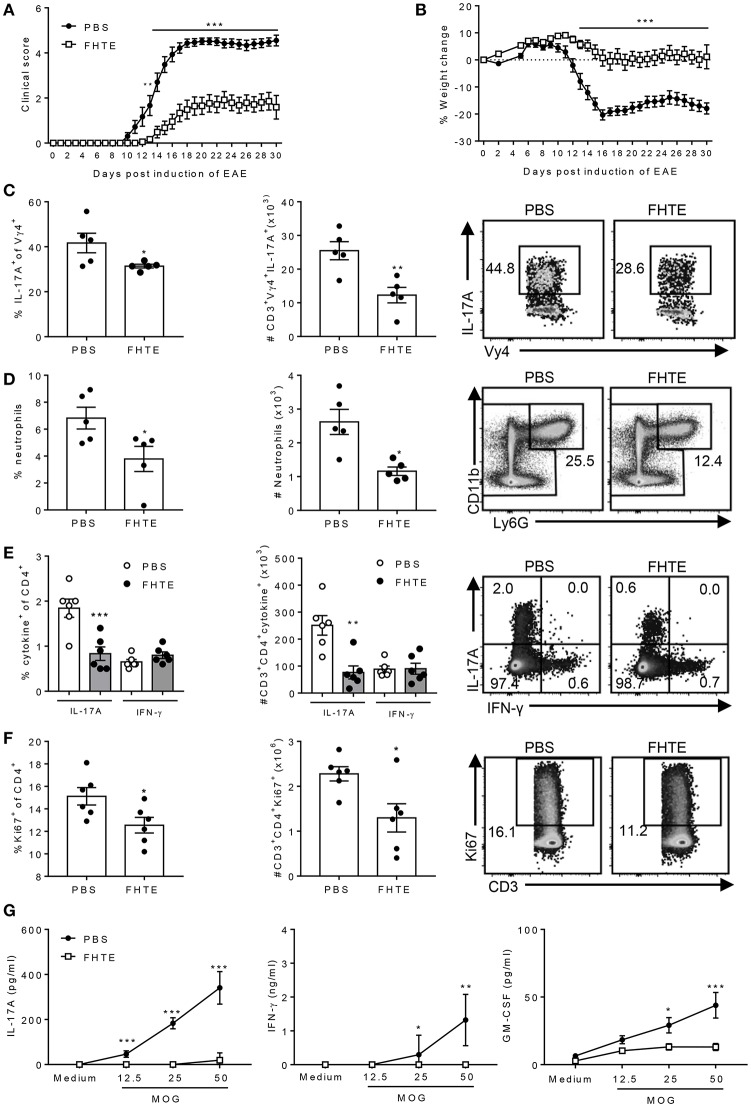
Pre-treatment of mice with FHTE suppresses induction of γδ T cell and Th17 cell responses and attenuates development of EAE. C57BL/6 mice were trained with PBS or FHTE (50 μg) 21 and 7 d prior to the induction of EAE. Mean clinical score **(A)** and weight changes **(B)** after induction of EAE. Results are mean ± SEM (*n* = 15–21) and data is combined from four independent experiments. ^**^p < 0.01, ^***^p < 0.001 vs. PBS-injected mice by repeated measures ANOVA with Sidak *post hoc* analysis. **(C)** LN cells were stained for surface TCR-δ and Vγ4 and intracellular IL-17A on d 3 of EAE and analyzed by flow cytometry, with sample FACS plots. **(D)** Spleen cells were stained for CD11b and Ly6G on d 3 of EAE and analyzed by flow cytometry, with sample FACS plots. **(E,F)** LN cells were stained for surface CD3 and CD4, intracellular IL-17A and IFN-γ and intranuclear Ki67 on d 7 of EAE and analyzed by flow cytometry, with sample FACS plots. **(E)** Frequency and absolute number of IL-17A and IFN-γ-producing CD4 T cells and **(F)** percentage and absolute number of Ki67^+^ CD4 T cells with sample FACS plots. Results are mean ± SEM (*n* = 5 or 6) and represent one of three independent experiments. Each symbol represents an individual mouse. ^*^p < 0.05, ^**^p < 0.01, ^***^p < 0.001 vs. PBS-injected mice by unpaired *t*-test. **(G)** Spleen and LN cells from d 7 of EAE were stimulated for 72 h with MOG peptide (0–50 μg/ml) and IL-17A, GM-CSF and IFN-γ concentrations in supernatants were determined by ELISA. Results are mean ± SEM (*n* = 6 mice) and represent one of three independent experiments. ^*^p < 0.05, ^**^p < 0.01, ^***^p < 0.001 vs. PBS-injected mice by two-way ANOVA with Sidak *post hoc* analysis.

Our results have demonstrated that innate immune training with FHTE promotes the generation of AAMs that induce production of the anti-inflammatory cytokines IL-10 and IL-1RA that can suppress pathogenic T cells that mediate EAE. Next, we examined the possibility that innate immune training of mice with helminth products could reduce their susceptibility to the development of EAE. Mice were pre-treated by two single injections of FHTE 21 and 7 d before induction of EAE. We found that clinical course of EAE was significantly attenuated in mice treated with FHTE (Figure 6A). Furthermore, mice injected with PBS lost a significant amount of weight during the effector phase of disease, whereas no weight loss was evident in mice treated with FHTE, reflecting the attenuated disease in these mice (Figure 6B).

The development of EAE involves peripheral activation of innate immune cells, including DCs and macrophages that produce IL-1β and IL-23, which promotes early IL-17A production by γδ T cells, especially Vγ4 T cells. This leads to recruitment of neutrophils and inflammatory monocytes, which activates the cascade leading to the induction of encephalitogenic Th17 cells (33, 34). Here we found that the frequency and absolute number of IL-17A-producing Vγ4 γδ T cells in the LNs was significantly reduced in mice treated with FHTE when compared with PBS-treated mice on d 3 after the induction of EAE (Figure 6C). Furthermore, neutrophil recruitment was reduced in the spleen of mice treated with FHTE when compared with PBS-injected mice on d 3 after the induction of EAE (Figure 6D).

Peripheral CD4 T cell activation and expansion were also significantly attenuated in the LNs of mice treated with FHTE on d 7 after the induction of EAE. The number of IL-17A-producing CD4 T cells was significantly reduced in mice treated with FHTE when compared with PBS-injected mice (Figure 6E). Furthermore, the frequency and absolute number of Ki67^+^ proliferating CD4 T cells was significantly decreased in the LNs of mice treated with FHTE (Figure 6F). An examination of MOG-specific T cell responses in the LNs 7 d after induction of EAE revealed that IL-17A, IFN-γ, and GM-CSF production was significantly reduced in mice treated with FHTE (Figure 6G). These results indicate that pre-treatment of mice with FHTE suppresses the induction and expansion of autoantigen-specific pathogenic T cells.

### Pre-treatment of Mice FHTE Reduces Infiltration of Neutrophils, Inflammatory Monocytes, γδ T Cells and Th17 Cells Into the CNS of Mice With EAE

**Figure 7:**
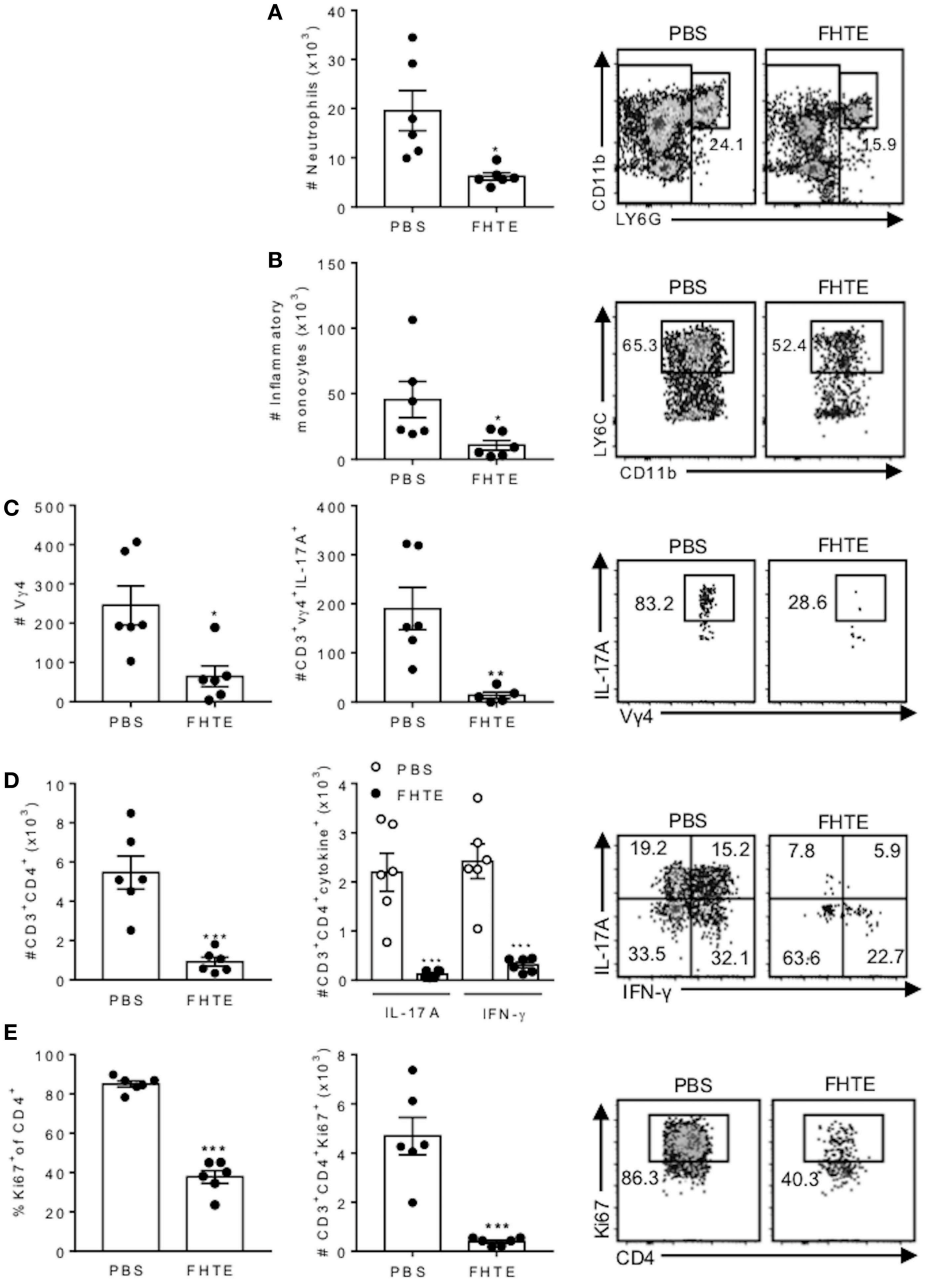
Reduced infiltration of neutrophils, inflammatory monocytes, γδ T cells and Th17 cells into the CNS of mice pre-treated with FHTE 12 d post EAE. Mice were treated with PBS or FHTE and EAE induced as described in Figure 5. **(A–D)** Flow cytometric analysis was performed on brain infiltrating cells of mice 12 d post induction of EAE. Absolute numbers of neutrophils **(A)** and inflammatory monocytes **(B)** in the brain on d 12 of EAE, with sample flow plots. **(C)** Absolute numbers of infiltrating Vγ4 T cells and absolute number of IL-17A-producing Vγ4 T cells, with sample FACS plots. **(D)** Absolute numbers of infiltrating CD4 T cells and CD4 T cells that secrete IL-17A or IFN-γ, with sample FACS plots. **(E)** Frequency and absolute number of Ki67^+^ proliferating CD4 T cells, with sample FACS plots. Results are mean ± SEM (*n* = 5–6) and represent one of three independent experiments. Each symbol represents an individual mouse. ^*^p < 0.05, ^**^p < 0.01, ^***^p < 0.001 vs. PBS-injected mice by unpaired *t-*test.

The CNS pathology in EAE is mediated by infiltration of encephalitogenic T cells and myeloid cells (35). Although the peak of disease is around d 16, our previous studies have demonstrated that the peak of CNS infiltrating T cells precedes the peak of disease. Therefore, we analyzed infiltration of cells into the brain on d 12 post induction and found that the absolute number of neutrophils and inflammatory monocytes infiltrating the brain was significantly reduced in mice treated with FHTE, when compared with PBS-injected mice (Figures 7A,B). Compared with PBS-injected mice, mice treated with FHTE also had significantly reduced numbers of infiltrating Vγ4 γδ T cells and IL-17A-producing Vγ4 γδ T cells (Figure 7C). Furthermore, mice treated with FHTE also had a significant reduction in the total number of CD4 T cells, as well as IL-17A, IFN-γ or IL-17 and IFN-γ co-producing CD4 T cells in the brain on d 12 (Figure 7D). In addition, the frequency and number of Ki67^+^ proliferating CD4 in the brain on d 12 of mice treated with FHTE was significantly diminished (Figure 7E). These results demonstrate that pre-treatment of mice with FHTE reduces their susceptibility to the development of EAE by suppressing the induction, migration and effector function of encephalitogenic IL-17-secreting γδ T cells and Th17 cells, through anti-inflammatory trained immunity.

## Discussion

The significant new findings of this study are that the innate immune system can, through epigenetic modifications, be trained to be more anti-inflammatory, thereby reducing the host's capacity to mount pro-inflammatory immune responses that mediate autoimmunity. Previous studies have reported that fungal products can train human monocytes to be pro-inflammatory, producing more TNF and IL-1 in response to a secondary stimulus (5, 30). However, our study demonstrates for the first time that macrophages can be trained with helminth products for heightened production of IL-10 and IL-1RA, while suppressing innate pro-inflammatory cytokines and Th1 and Th17 responses that mediated CNS inflammation in the EAE model. Our findings suggest priming of the innate immune system with the appropriate environmental cues can have protective influence against the development of T cell-mediated autoimmune diseases.

Immunological memory was traditionally thought to be confined to the adaptive immune system, however, recent studies have shown that the innate immune system has a type of memory or can be trained (36, 37). Immunological imprinting through tolerance or trained immunity influences the capacity of the immune system to respond to a secondary stimulus or infection (38). Consistent with previous reports, we found that training of macrophages with LPS induced unresponsiveness to subsequent TLR activation. In contrast, training of macrophages with β-glucan enhanced their responsiveness to stimulation with TLR agonists. The latter finding is consistent with a study by Saeed et al. who demonstrated that exposure to certain microbial stimuli increases long-term responsiveness of monocytes to secondary infection (5). The present study revealed that microbial stimuli from helminth pathogens can promote an alternative form of innate immune training that results in heightened anti-inflammatory responses. Training of macrophages with FHTE increased secretion of IL-1RA and IL-10, while inhibiting TNF production in response to restimulation with Pam3Cys or LPS. Although we do not know the persistence of this training effect induced with FHTE, we found that innate training effect of *F. hepatica* excreted/secreted products was maintained for several months through modulation of macrophage precursors in the bone marrow (Cunningham and Mills, unpublished data).

Previous studies in trained immunity have shown that the enhanced responsiveness of monocytes or macrophages to subsequent encounters with a pathogen or PAMPs was mediated by epigenetic changes (39). Monocytes/macrophages trained *in vitro* or *in vivo* with BCG or β-glucan produced higher concentrations of pro-inflammatory cytokines than untrained cells following restimulation with inflammatory PAMPS and training was reversed in the presence of epigenetic inhibitors (5, 40). The significant advance in our study is the demonstration that innate immune cells can be trained to be more immunosuppressive following exposure to helminth products and that this anti-inflammatory training effect appears to be mediated by epigenetic modification of macrophages. In contrast to previous studies on innate training, we have shown that trained immunity with the appropriate stimuli can result in reduced inflammatory, as well as enhanced anti-inflammatory, cytokine production in response to inflammatory PAMPs.

There are several reports that helminths can modulate innate immune responses, including DC and macrophages and/or attenuate T cell responses that promote autoimmune diseases (13–15, 25, 41, 42). However, most studies on helminth product modulation of DCs or macrophages directly treated the cells and immediately assessed cytokine production, with or without TLR activation (9, 10, 43–45). There is one report that the parasite *Plasmodium falciparum* can induce innate immune training, however, this parasite enhanced the pro-inflammatory response of PBMCs (36). We showed that macrophages that had been treated with FHTE, rested and then stimulated with a TLR agonists had a trained anti-inflammatory phenotype. Furthermore, in most previous studies on modulation of autoimmune diseases *in vivo* that we are aware of, the mice were “treated” with helminth products at disease onset and/or during disease. Rather than injecting the mice with helminth products at induction or during development of autoimmunity, the mice by pre-treatment with two single injections, rested and as a result they were less susceptible to induction of EAE. Treatment of mice by two single injections of FHTE led to the accumulation of macrophages in the peritoneal cavity and these cells had reduced pro-inflammatory but enhanced anti-inflammatory cytokine production when re-stimulated with different TLR ligands *ex vivo*. Furthermore, we show that macrophages from the FHTE-treated mice were immunosuppressive *in vitro* and *in vivo* when transferred to mice with EAE.

Interestingly immunization of infants with the BCG vaccine has been shown to reduce the incidence of unrelated infectious diseases and this is mediated not through classical adaptive memory but through innate immune memory or trained immunity, where the innate immune cells mount more effective inflammatory responses to the unrelated pathogen (46). Interestingly, there is also evidence that certain infections or sterile inflammation, triggered by inflammatory DAMPs or alarmins, may trigger autoimmune diseases by heightening innate and consequently self-antigen-specific adaptive immune responses (47). Our study has revealed a reciprocal mechanism, whereby exposure to certain agents, such as helminth products, reduces the capacity of innate immune cells to respond to inflammatory stimuli that might otherwise precipitate autoimmunity. This may explain the reduced risk of developing autoimmune disease in individuals infected with helminth parasites.

Treatment of mice with FHTE was accompanied by enhanced expression of CD206 and PD-L2 on SPM, markers consistent with the polarization of AAM during helminth infection (48). Although it is not clear that there are distinct M1 and M2-type macrophages *in vivo*, the present study demonstrated that SPM from mice treated with FHTE expressed high levels of M2-associated genes *arg1* (encoding arginase 1) and *retlna* (encoding relmα). Relmα has been shown to suppress inflammation and helminth-induced Th2-type immunity (49). The induction of arginase expression likely reflects wound repair mechanisms during prolonged helminth infection (50). Furthermore, arginase produced by AAM depletes L-arginine from the tissue microenvironment, which inhibits T-cell function (51). In addition, mice treated with FHTE also had increased expression of M2-associated gene *mrc1* (encoding CD206) and *chi3l*3 (encoding YM1) in SPM. This is consistent with a report demonstrating enhanced expression of *chi3l3* in response to *Schistosoma mansoni* infection, which is important for limiting parasite survival (52). It has also been reported that long-term infection with the helminth parasite *Taenia crassiceps* induces monocyte-derived AAM that attenuate EAE (48). However, the present study is the first to demonstrate anti-inflammatory training of macrophages; two single exposures to FHTE suppressed the activation of encephalitogenic T cells responses and the development of EAE. Therefore, polarizing AAM may be an important mechanism evolved by helminths to subvert immune responses and their expulsion from the host, but training of macrophages by helminths to adopt an AAM phenotype may have wider consequences in reducing the susceptibility to autoimmune diseases.

Our findings demonstrate that training of macrophages to be anti-inflammatory through exposure to helminth-derived products can attenuate induction of EAE, a disease that can be mediated exclusively by T cells. The pathology of EAE is mediated by peripherally-induced innate γδ T cells and autoantigen-specific Th1 and Th17 cells that promote inflammation following migration into the CNS (53). The activation of γδ T cells and Th17 cells is dependent on innate sources of IL-1β and IL-23. Mice defective in IL-1R1 or mice treated with inhibitors of IL-1 production are resistant to EAE (33), whereas IL-1RA can suppress the effect of IL-1β and IL-1α on IL-17 secretion by T cells (54). We found that FHTE induced IL-10 and IL-1RA production, while suppressing TLR-induced production of TNF and IL-12p40 by macrophages. Furthermore, macrophages from mice trained *in vivo* with FHTE suppressed IL-17 production by MOG-specific T cells. This was reflected in significant suppression of IL-17A-producing Vγ4 T cells and CD4 T cells and attenuation of disease in the EAE model. Therefore, the mechanism of attenuation of autoimmune disease in mice treated with FHTE involves trained macrophage suppression of pathogenic T cell responses.

Several studies in mouse models of autoimmunity have shown that infection with helminth parasites can attenuate autoimmune disease (15, 17, 41, 55). Our study has extended these findings by demonstrating that two single exposures of mice to products of the helminth *F. hepatica* can train innate immune cells to be more anti-inflammatory and thereby reduce their susceptibility to the induction of autoimmune inflammation. Although *F. hepatica* can infect humans, infection with other helminths, such as Schistosomes, or nematodes, such as Ascaris or Trichuris, are likely to have a greater impact on human autoimmune diseases (56). Indeed, it has already been reported that MS patients with helminth infection have reduced number of relapses and better disease outcome than non-infected patients (24). Our study suggests that epigenetic modification of macrophages by helminths products that renders them immunosuppressive may in part may explain the reduced susceptibility to autoimmune diseases in individuals living in areas with high helminth burden. Importantly, it also identifies a novel phenotype of innate training involving anti-inflammatory innate immune responses.

## Data Availability

The datasets generated for this study are available on request to the corresponding author.

## Ethics Statement

All mice were maintained according to European Union regulations, and experiments were performed under license (AE19136/P042) from the Irish Health Products Regulation Authority with approval from the Trinity College Dublin BioResources Ethics Committee.

## Author Contributions

SQ designed and performed experiments, analyzed data, and co-wrote the manuscript. KC, MR, RW, LC, and AM performed experiments. KM conceived ideas, oversaw the project and co-wrote the manuscript.

### Conflict of Interest Statement

The authors declare that the research was conducted in the absence of any commercial or financial relationships that could be construed as a potential conflict of interest.

## Supplementary Material

Supplemental Figure 1 Cytokine production by macrophages in responses to a single stimulation with FHTE, LPS or β-glucan. BMDMs were incubated with 2.5% v/v FHTE, LPS (100 ng/ml) or β-glucan (5 μg/ml). Cells were washed after 24 h and after 3 d rest, supernatants were collected and the concentrations of IL-1RA **(A)**, IL-10 **(B)**, and TNF **(C)** was determined by ELISA. Results are mean ± SED combined from three experiments. ^***^*p* < 0.001 vs. medium cultured cells by one-way ANOVA with Dunnett *post hoc* analysis.

## Abbreviations

AAM: alternatively activated macrophages
BCG: bacille Calmette-Guerin
BMDMs: bone narrow-derived macrophages
DCs: dendritic cells
EAE: experimental autoimmune encephalomyelitis
FHTE: Fasciola hepatica total extract
LN: lymph node
MOG: myelin oligodendrocyte glycoprotein
PEC: peritoneal exudate cell
SPM: small peritoneal macrophage
Treg cell: regulatory T cell.
