# Supplementary material for: Anti-inflammatory Trained Immunity Mediated by Helminth Products Attenuates the Induction of T Cell-Mediated Autoimmune Disease
Source: Front Immunol. 2019 May 21;10:1109. doi: 10.3389/fimmu.2019.01109 (PMC6537856; doi:10.3389/fimmu.2019.01109)
Supplement: Supplemental Figure 1 — Cytokine production by macrophages in responses to a single stimulation with FHTE, LPS or β-glucan. BMDMs were incubated with 2.5% v/v FHTE, LPS (100 ng/ml) or β-glucan (5 μg/ml). Cells were washed after 24 h and after 3 d rest, supernatants were collected and the concentrations of IL-1RA (A), IL-10 (B), and TNF (C) was determined by ELISA. Results are mean ± SED combined from three experiments. ***p < 0.001 vs. medium cultured cells by one-way ANOVA with Dunnett post hoc analysis. [file Image_1.pdf]

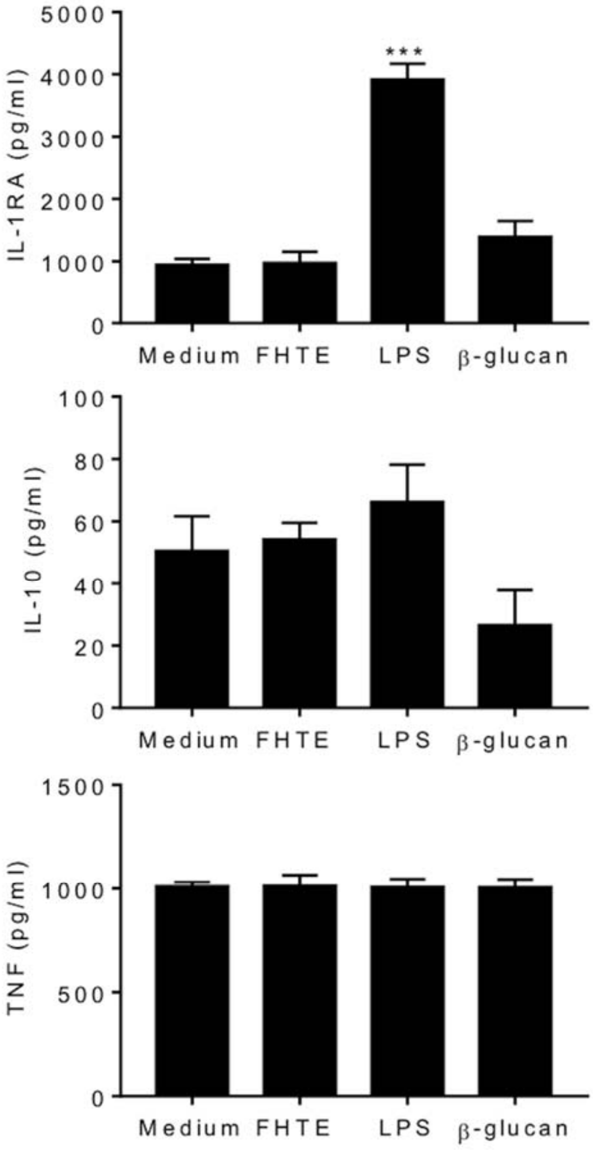

Supplemental figure 1. Cytokine production by macrophages in responses to a single stimulation with FHTE, LPS or β-glucan. BMDMs were incubated with 2.5% v/v FHTE, LPS (100 ng/ml) or β-glucan (5 μg/ml). Cells were washed after 24 h and after 3 d rest, supernatants were collected and the concentrations of IL-1RA (**A**), IL-10 (**B**) and TNF (**C**) was determined by ELISA. Results are mean ± SED combined from three experiments. ***$p<0.001$ vs medium cultured cells by one-way ANOVA with Dunnett post hoc analysis.
